# Implementation of improvement strategies in palliative care: an integrative review

**DOI:** 10.1186/s13012-015-0293-2

**Published:** 2015-07-26

**Authors:** Jasper van Riet Paap, Myrra Vernooij-Dassen, Ragni Sommerbakk, Wendy Moyle, Marianne J. Hjermstad, Wojciech Leppert, Kris Vissers, Yvonne Engels, on behalf of the IMPACT research team

**Affiliations:** Scientific Institute for Quality of Healthcare (IQ healthcare), Radboud University Medical Center, P.O. Box 9101, 6500 HB Nijmegen, The Netherlands; Nijmegen Alzheimer Centre, Radboud University Medical Center, P.O. Box 9101, 6500 HB Nijmegen, The Netherlands; Kalorama Foundation, Nijmegen, The Netherlands; European Palliative Care Research Centre, Department of Cancer Research and Molecular Medicine, Faculty of Medicine, Norwegian University of Science and Technology, P.O. Box 8905, N-7491 Trondheim, Norway; Centre for Health Practice Innovation, Griffith Health Institute, Griffith University, 170 Kessels Road, Nathan, Brisbane Australia; Regional Centre for Excellence in Palliative Care Department of Oncology, Oslo University Hospital, P.O. Box 4956, Nydalen, 0424 Oslo Norway; Department of Palliative Medicine, Poznan University of Medical Sciences, 61-245 Poznan, Poland; Department of Anaesthesiology, Pain and Palliative Medicine, Radboud University Medical Center, P.O. Box 9101, 6500 HB Nijmegen, The Netherlands

**Keywords:** Palliative care, Europe, Implementation, Improvement strategies

## Abstract

**Background:**

The European population is ageing, and as a consequence, an increasing number of patients are in need of palliative care, including those with dementia. Although a growing number of new insights and best practices in palliative care have been published, they are often not implemented in daily practice. The aim of this integrative review is to provide an overview of implementation strategies that have been used to improve the organisation of palliative care.

**Methods:**

Using an integrative literature review, we evaluated publications with strategies to improve the organisation of palliative care. Qualitative analysis of the included studies involved categorisation of the implementation strategies into subgroups, according to the type of implementation strategy.

**Results:**

From the 2379 publications identified, 68 studies with an experimental or quasi-experimental design were included. These studies described improvements using educational strategies (*n* = 14), process mapping (*n* = 1), feedback (*n* = 1), multidisciplinary meetings (*n* = 1) and multi-faceted implementation strategies (*n* = 51). Fifty-three studies reported positive outcomes, 11 studies reported mixed effects and four studies showed a limited effect (two educational and two multi-faceted strategies).

**Conclusions:**

This review is one of the first to provide an overview of the available literature in relation to strategies used to improve the organisation of palliative care. Since most studies reported positive results, further research is needed to identify and improve the effects of strategies aiming to improve the organisation of palliative care.

**Electronic supplementary material:**

The online version of this article (doi:10.1186/s13012-015-0293-2) contains supplementary material, which is available to authorized users.

## Background

The European population is ageing, and as a consequence, an increasing number of patients are in need of palliative care, including those with dementia. The World Health Organization has defined palliative care as an ‘approach to improve the quality of life of patients and families who face life-threatening illness, by providing pain and symptom relief, spiritual and psychosocial support from diagnosis to the end of life and bereavement’ [1–3]. Although a growing number of new insights and best practices in palliative care are being published, knowledge translation into daily practice is lacking [4]. Study results in both the USA and the Netherlands suggest that up to 40 % of patients in need of palliative care do not receive evidence-based care [5]. Apparently, there is a wide ‘gap’ between the available scientific evidence and its use in daily practice [5].

The implementation of new evidence into daily practice is particularly challenging when complex changes are needed, cooperation between disciplines is required, or behaviour needs to be changed [6]. The use of traditional implementation strategies to convince professional care providers to use new evidence (such as identifying, synthesizing and disseminating evidence in journals, guidelines, continuing medical education and conferences) is apparently not sufficient to engineer changes in the complex systems of palliative care [5].

Yet, many studies that aim to improve palliative care have been performed. Often, these studies require much time investment and money from both the professional workforce as well as patients, which raises cost-effectiveness questions. It is therefore of utmost importance to synthesise and disseminate state-of-the-art scientific knowledge [7, 5]. The aim of this integrative review is to provide an overview of effective implementation strategies that have been used to improve the organisation of palliative care. As such, results of this review have been used in the EU-funded Seventh Framework IMPACT project (*IM*plementation of quality indicators for *PA*lliative *C*are s*T*udy) which aims to develop and tailor national and setting-specific strategies to improve the organisation of palliative care in Europe [8].

## Methods

A review of available research literature was considered important to identify current knowledge about this topic.

The integrative review methodology summarizes past empirical and theoretical literature that uses diverse methodologies and study designs from a variety of sources in order to provide a comprehensive understanding of a complex health care problem [9]. Therefore, an iterative comparison and analysis of relevant publications about the implementation of strategies to improve the organisation of palliative care was conducted.

### Search strategy

A comprehensive literature review was conducted, including Medline, CINAHL, British Nursing Index, PsycINFO, and by searching for grey literature [10] (e.g. literature that has not been published in peer-reviewed literature). The search strategy was limited to English literature only, to publications that concerned palliative care for adults (aged 18 or above) and to publications that were published between 2000 and August 2011. Various search terms were used that referred to subject-specific keywords describing palliative care, as well as the type of implementation strategy and outcomes of the implementation, including synonyms and Medical SubHeadings (MeSH) to include all relevant literature. Table 1 provides an overview of the search terms (the search string is available in Additional file 1). Disease-specific search terms, such as cancer or dementia, were not included because the organisation of palliative care goes beyond a specific disease [11].

**Table 1:** Overview of search terms

Palliative care	Implementation strategy	Outcomes of implementation
Terminal care	Health plan implementation	Quality of health care
Hospices	Programme development	Programme evaluation
Hospice care	Quality indicators	Quality
End-of-life care	Implementation strategy	Improvement
Comfort care	Programme evaluation	Change
Supportive care	Information dissemination	
Cancer care facilities	Information distribution	
Oncology service, hospital	Organisational innovation	
	Organisation change	
	Diffusion of innovation	
	Educational models	
	Organisational models	
	Quality improvement	

### Inclusion and exclusion criteria

Publications were included when they described (1) improvements to the organisation of palliative care, (2) which implementation strategies were used, (3) how these strategies were implemented and (4) the effectiveness of these strategies. Publications were excluded when (1) no abstract was available, (2) the implementation strategies were not directed at health care professionals or volunteers, (3) they were not directed at adult health care services or (4) educational curricula were developed.

### Data extraction and analysis

Two of the authors (JvRP and RS, one with a background in nursing and health sciences and the other in sociology) independently screened title and abstract and reviewed the full-text articles of the included studies to identify implementation strategies to improve the organisation of palliative care. A data collection form was used to extract information about the country and year in which the study was published, study design, setting, type of disease, health care professionals involved, and type, description and impact of the implementation strategies used. Subsequently, implementation strategies were categorised into subgroups, according to the type of implementation strategy, similar to the approach of Grol and Grimshaw [5]. Data from the subgroup classification was coded and compiled into a matrix, whereby the effect of each implementation strategy was summarized as a significant improvement (++), improvement (+), mixed or limited effect (+/−) or no effect (−). To ensure the trustworthiness and rigor of the analysis, peer debriefing took place with the other authors throughout the entire process of data analysis.

## Results

### Study selection

**Fig. 1:**
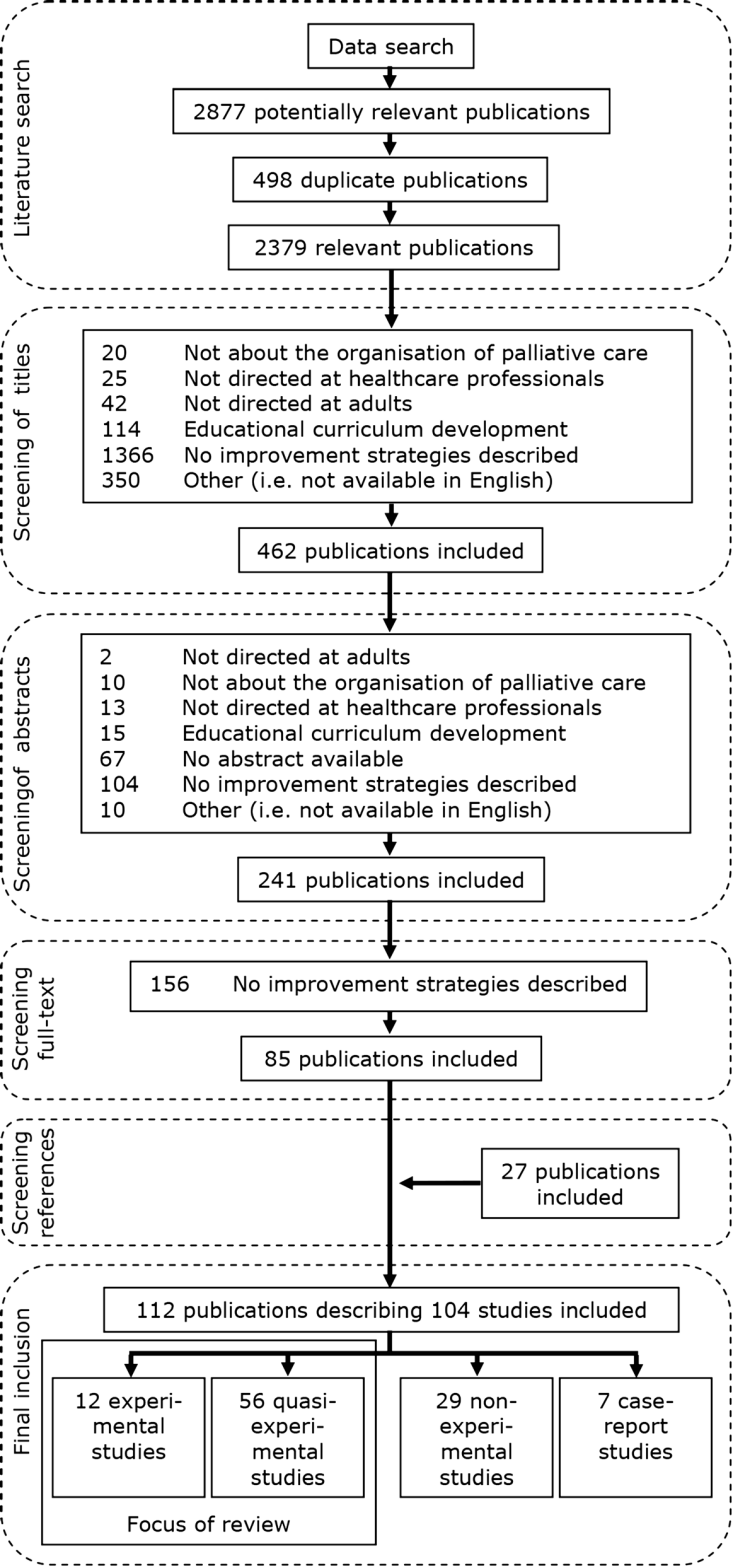
Flow chart

Of the 2379 initially identified publications, 241 were selected for full-text assessment (Fig. 1). A first assessment of the full-text of these publications revealed that 156 publications could be excluded, as they did not meet the inclusion criteria (for example, because there were no details given about the improvement strategy used). Reference lists of all publications eligible for inclusion as well as a hand search in grey literature databases revealed an additional 27 publications eligible for inclusion. The remaining publications represented a wide variety of research methods and designs: interviews, focus groups, strategy development designs, case descriptions, surveys, process evaluations, RCTs, pre-post-test interventions, review papers as well as theoretical papers. Because of the large number of identified studies, of which many were of low scientific quality and with incomparable outcome measures, only studies with an experimental (*n* = 12) or quasi-experimental (*n* = 56) study design were selected for further analysis.

### Characteristics of included studies

A total of 17 single intervention studies and 51 multi-faceted intervention studies were identified. Most studies were conducted in the USA (*n* = 29), UK (*n* = 19) and Australia (*n* = 8), but studies were also included from countries such as Japan, Taiwan, Italy and the Netherlands. Studies were conducted within the entire range of palliative care services, from home care services to advanced palliative care units in hospitals. Fifty-one studies were conducted in one setting (primary care: *n* = 2, hospital: *n* = 38, nursing home: *n* = 9, hospice: *n* = 1 and other: *n* = 1) and 11 were conducted in multiple settings. For six studies, the type of setting could not be identified. Within the included studies, a large variety of professionals participated. Thirty-five studies were directed at a single type of professionals (e.g. nurses only), 29 at two or more different groups of professionals (e.g. nurses and physicians), and four studies did not report the target group of professionals.

### Strategies and its impact

Additional file 2 provides a summarized description of the methodology, setting and country, number and type of participants, the implementation strategy and the impact of the strategy of each individual study. Table 2 provides an overview of the results of studies with an experimental or quasi-experimental study design.

**Table 2:** Effect of strategies specified to setting

Strategy		No.	Primary care	Hospital	Nursing home	Hospice	Other, multiple or unknown settings
Single intervention studies	Lecture	3		Ke, 2008 (++)		Schim, 2006 (+)	Ersek, 2006 (++)
	Study day	2					Carr, 2003 (+); Dryden, 2009 (+)
	Role play	2		Hales, 2008 (+)			Back, 2007 (+)
	Interactive education	2		Bruneau, 2004 (−); Cooke, 2004 (+)			
	Outreach visit	1					Newton, 2009 (+)
	Computer-facilitated education	4		Hulsman, 2002 (−); Jarabek, 2008 (++)			Ersek, 2008 (++); Smith, 2010 (+)
	Process mapping	1			Taylor, 2007 (+)		
	Feedback	1		Velikova, 2004 (+)			
	Multidisciplinary meetings	1		Lilly, 2000; Lilly, 2003 (++)			
Multi-faceted intervention studies	Multi-educational interventions	25	Reymond, 2005 (++)	Bylund, 2010 (+/−); Furman, 2006 (+/−); Betcher, 2010 (+); Hall, 2007 (+); Kinnane, 2009 (+); Razavi, 2002 and Delvaux, 2004 (+); Fischer, 2007 (++); Kruse, 2008 (++); Bailey, 2005 (++); Fallowfield, 2001 (++); Gueguen, 2009 (++);Sutherland, 2007 (++); Yamagishi, 2009 (++)	Weissman, 2000 (+); Weissman, 2001 (+)		Bravemen, 2001 (+/−); Razavi, 2003 and Lienard, 2006 and Lienard, 2008 and Merckaert 2008 (+/−); Wilkinson, 2008 (+); Finset, 2003 (+); Favre, 2007 (++); Quinn, 2008 (++); Sullivan, 2005 (++); Wilkinson, 2002 (++); Wilkinson, 2003 (++)
	Mixed interventions	26	Boakes, 2000 (++)	Butow, 2008 (−); Curtis, 2011 (−); Fallowfield, 2002 and Jenkins, 2002 and Shilling, 2003 and Fallowfield, 2003 (+/−); Hansen, 2009 (+/−); Hills, 2009 (+/−); Jacobs, 2002 (+/−); McCormick, 2010 (+/−); Morgan, 2010 (+/−); Roila, 2004 (+/−); Kinley, 2004 (+); Lankshear, 2010 (+); Mirando, 2005 (+); Monteleoni, 2004 (+); Okon, 2009 (+); Smith, 2009 (+); Bookbinder, 2005 (++); Dauer, 2006 (++)	Hanson, 2005 (+); Hockley, 2010 (+); Lyon, 2007 (+); Reynolds, 2004 (+); Strumpf, 2004 (+); Keay, 2003 (++)		Stacey, 2008 (+); Woo, 2011 (+)

*++* significant improvement, *+* improvement, *+/−* mixed or limited effect, *−* no effect

### Educational strategies

Two experimental and 12 quasi-experimental studies used different types of educational strategies to improve palliative care, including lectures [12–14], study days [15, 16], role play sessions [17, 18], interactive education [19, 20], educational outreach visits [21] and computer-facilitated education [22–25]. Eight studies were targeted at a single profession, while six were targeted at multiple professionals. Four studies reported significant improvements, eight reported improvement, and two studies had limited or no effect.

### Process mapping

One study used process mapping to improve the organisation of palliative care in a nursing home [26]. Before implementing the Liverpool Care Pathway, nursing home staff organized interdisciplinary team discussions where they answered the question, ‘If your patient is diagnosed as dying at 10 am on Monday morning and they are in pain, what happens?’ or in other words, ‘What is the process?’ in order to identify bottlenecks to be expected [26]. A repeated process measure post-implementation reduced the numbers of expected bottlenecks.

### Feedback

One study addressed feedback to improve the organisation of palliative care [27]. In an RCT, patients completed a health-related quality of life questionnaire. In the intervention group, hospital physicians received automated feedback upon completion of this questionnaire. The RCT showed improved health-related quality of life in the intervention group compared to the control group.

### Multidisciplinary meetings

One quasi-experimental study performed by Lilly et al. described family and multidisciplinary meetings to improve the communication and shared-decision making at the intensive care unit (ICU) in a hospital [28, 29]. Pre- and post-intervention measurements showed that the use of such meetings reduced length of ICU stay.

### Mixed interventions

Fifty-one studies used a combination of strategies. Half of these studies used a combination of solely educational strategies (for example, lecture combined with role play sessions) [30–58], while the other half combined a variety of strategies (for example, education combined with feedback and reminders) [59–86]. Nine studies had an experimental design. Based on the conclusions of the authors, there were 13 studies that had no or a limited effect on the specified outcomes, 21 studies that had a positive effect, and the remaining 17 studies reported a significant improvement.

## Discussion

We made a comprehensive overview of the available literature in relation to strategies used to improve the organisation of palliative care. In total, 68 studies, representing an experimental or quasi-experimental study design, were discussed. These studies included educational strategies, process mapping, feedback, multidisciplinary meetings as well as mixed interventions.

For this review, all reported outcome measures in the identified studies were extracted. These measures included several patient outcome data items (e.g. assessment of the percentage of patients in pain following an educational session about pain treatment) as well as data concerning the process of care (e.g. the frequency of patient referral to specialist care following the introduction of a new referral form), making comparisons of outcomes impossible. However, the aim of all included studies was to improve the organisation of palliative care. We therefore generalized the outcomes to the degree in which they aimed to improve the organisation of palliative care.

Fifty-three studies, covering all strategies identified, reported that their study resulted in improving the organisation of palliative care. Eleven studies showed improvements for some of the characteristics targeted (four multi-educational and seven multi-faceted strategies), and four studies reported limited or no improvements (one using interactive education, one using computer-facilitated education and two multi-faceted strategies). The studies with a mixed or limited result on improving the organisation of palliative care were primarily conducted in one setting (hospital) and directed at one professional group. This stipulates the challenges that are encountered when implementing new evidence in complex environments such as a hospital as well as the importance of the multidisciplinary and interdisciplinary character of palliative care.

The different strategies identified in this review have been described in other fields in health care. In a review by Grol and Grimshaw, for example, large conferences and courses showed mixed effects, small group interactive education showed positive effects, educational outreach showed positive effects, feedback showed mixed effects and the used of mixed interventions often resulted in better results compared to single intervention studies [5]. Reviews that focused on one strategy type, for example, on audit and feedback [87], printed educational materials [88] or educational outreach visits [89], all had similar findings. One reason for the primarily positive findings of the studies identified in this review might be the fact that participants in a quality improvement project perform better as a result of knowing they are a study object (Hawthorne effect) [90]. Another reason might be that effects were often measured immediately after the intervention, so we do not know if the effects were sustained. Finally, only a few studies (*n* = 12) used a randomised controlled design, which is often considered to be the gold standard in research [91], compared to other designs. RCTs require significant time and funding and expert research guidance, and particularly, in palliative care populations, they are scarce because of recruitment restrictions, high attrition, (selection) bias, lack of blinding, confounding and small sample sizes [92, 91]. Many of these aspects, however, are also relevant in studies with a quasi-experimental, non-experimental or case-study design. The studies that were included in this review might, therefore, not represent the strongest designs to test improvement strategies.

The included studies were conducted in a variety of settings (e.g. hospitals, nursing homes, hospices and primary care facilities). The provision of palliative care within these settings may vary depending on the patient group. Patients with cancer, for example, have a different disease trajectory, and other symptoms and needs than persons with dementia [93, 94]. Despite these differences, there are many similarities regarding the organisation and multidisciplinary character of palliative care. For all chronic, life-threatening conditions, palliative care entails a patient-centred approach in which multidimensional interventions related to actual and future problems, needs and preferences are made.

The WHO definition of palliative care is therefore applicable for all patient groups [3]. The European Association of Palliative Care illustrates this by recommending a common approach for palliative care across settings [95, 96]. In addition, 40 international experts agreed that there is no need to formulate disease-specific quality criteria for the organisation of palliative care [97]. However, this does not mean that there is ample evidence regarding effective strategies to improve (the organisation of) palliative care in the different settings. Hall et al., for example, described that there is limited evidence for palliative care service delivery for residents of care homes for older people [98]. This illustrates the necessity to further improve the field of implementation science, in particular, in underdeveloped areas such as palliative care for persons with dementia.

Quality improvement projects often require investments of time and money from both the professional workforce and patients. It is important that the evidence of effective strategies is used to improve daily clinical practice. However, researchers and professionals often have different cultures, values, timelines, goals and rewards [99]. Even when the intervention is well-designed, real-world contextual factors may prevent the intervention from being realized. Implementation of evidence-based and best practices should therefore always be guided by a step-by-step model in order to identify the problem, barriers and facilitators and tailored strategies to solve the problem [100]. Integrated knowledge translation can then be used as a bridge in closing the gap between what we know and what we do [101].

The results of this review were used in the EU-funded Seventh Framework IMPACT project. An intervention study investigating improvement projects with pre- and post-test evaluations was performed in 40 services providing palliative care across Europe (including hospitals, nursing homes, hospices and primary care facilities). In this study, quality indicators were used to identify potential areas to improve the organisation of palliative care. Subsequently, Grol’s implementation of change model [100] was used to guide the services in their quality improvements. The strategies described in this review were used as an example and if possible also as actual strategies regarding how to change the organisation of palliative care.

### Strengths and limitations

This is one of the first reviews that provides an overview of implementation strategies used to improve the organisation of palliative care. The results of this review can be used as a starting point for further research. However, some limitations should be taken into account. Firstly, this review used the integrative review methodology. Although this approach allows for the combination of diverse methodologies (including non-experimental research), only studies with an experimental and quasi-experimental design were included because of the unexpected high number of publications on the highest evidence level. Since a variety of methods was used in these studies, a quantitative comparison of effect size was considered impossible. Secondly, because it was the aim of this review to provide an overview of strategies used to improve the organisation of palliative care rather than the effectiveness, we did not assess each individual study for risk of bias or effect estimates. The effects of the strategies presented in this paper should therefore be interpreted with caution. Thirdly, we have limited the search strategy to English literature only. Although publications have been included from non-English speaking countries such as Japan and Italy, it is likely that we have missed potentially interesting publications from countries that often publish in their own language. Fourthly, there is no generic set of search terms to identify literature about improvement strategies, despite the fact that improvements are now being recognized as a science [102, 103]. Although we have captured a broad selection of literature with our search strategy, it is possible that it did not identify all available publications on this topic.

## Conclusion

This review provides an overview of the available literature in relation to strategies used to improve the organisation of palliative care. The identified studies described educational strategies, process mapping, feedback, multidisciplinary meetings and multi-faceted interventions. Future research, with more rigid designs, proper duration, control and blinding are necessary to identify and improve scientific evidence regarding the optimal strategies to improve the organisation of palliative care.

## Additional files

Additional file 1:
**Search string.** The search strings for Medline, CINAHL, British Nursing Index and PsycINFO. (PDF 57 kb) Additional file 2:
**Description studies.** Summarized description of the methodology, setting and country, number and type of participants, the implementation strategy, and the impact of the strategy of each individual study. (PDF 182 kb)
