# Supplementary material for: Implementation of improvement strategies in palliative care: an integrative review
Source: Implement Sci. 2015 Jul 26;10:103. doi: 10.1186/s13012-015-0293-2 (PMC4515317; doi:10.1186/s13012-015-0293-2)
Supplement: Additional file 1: — Search string. The search strings for Medline, CINAHL, British Nursing Index and PsycINFO. (PDF 57 kb) [file 13012_2015_293_MOESM1_ESM.pdf]

Appendix 1: Search string

BNI
palliat*.ti,ab. or exp Terminal care/ or terminal care.ti,ab. or Terminal Care : Hospices/ or Terminal care : nursing/ or Terminal care : services/ or Cancer : services/ or hospic*.ti,ab. or end of life care.ti,ab. or comfort care.ti,ab. or supportive care.ti,ab.
AND
implement*.ti,ab. or program development.ti,ab. or "Standards and guidelines"/ or Audit/ or "Care plans and planning"/ or Health service planning/ or Management information systems/ or quality indicator*.ti,ab. or program evaluat*.ti,ab. or information disseminat*.ti,ab. or information distribut*.ti,ab. or organi?ational innovat*.ti,ab. or organi?ational chang*.ti,ab. or "Models and theories"/ or Quality assurance/
AND
Exp Quality assurance/ or quality*.ti,ab. or Management information systems/ or program evaluat*.ti,ab. or improv*.ti,ab. or chang*.ti,ab. or Evidence based practice/
Limitation
not (child* OR neonat* OR infant* OR pediatr* OR paediatr*).ti.
yr="2000 to current"

CINAHL
MH "Palliative Care" or TI palliat* OR AB palliat* OR MH "Terminal Care" or TI "terminal care" OR AB "terminal care" or MH "Hospice and Palliative Nursing" or MH "Hospices" or MH "Hospice Care" or MH "Hospice patients" or TI hospic* or AB hospic* or TI "end of life care" or AB "end of life care" or TI "comfort care" or AB "comfort care" or TI "supportive care" or AB "supportive care" or MH "Cancer Care Facilities"
AND
MH "Program development+" or MH "Patient care plans+" or TI "health plan implement*" or AB "health plan implement*" or TI "program implement*" or AB "program implement*" or TI "program development" or AB "program development" or MH "Clinical indicators" or TI "quality indicator*" or AB "quality indicator*" or TI "clinical indicator*" or AB "clinical indicator*" or MH "Quality improvement" or TI "quality improvement*" or AB "quality improvement*" or TI "implementation strateg*" or AB "implementation strateg*" or TI "program evaluat*" or AB "program evaluat*" or TI "information disseminat*" or AB "information disseminat*" or TI "information distribut*" or AB "information distribut*" or MH "Organizational change" or TI "organi?ational innovat*" or AB "organi?ational innovat*" or TI "organi?ational chang*" or AB "organi?ational chang*" or MH "Diffusion of innovation" or MH "Models, educational" or MH "Quality of care research"
AND
MH "Quality of Health Care+" or TI quality* or AB quality* or MH "Program Evaluation" or TI "program evaluat*" or AB "program evaluat*" or TI improv* or AB improv* or TI chang* or AB chang*
Limitation
not (MH child+ not MH adult+)
Published Date from: 20000101-20111231

MEDLINE
exp palliative care/ or palliat*.ti,ab. or terminal care/ or terminal care.ti,ab. or exp hospices/ or exp hospice care/ or hospic*.ti,ab. or end of life care.ti,ab. or comfort care.ti,ab. or supportive care.ti,ab. or exp cancer care facilities/ or exp oncology service, hospital/
AND
exp health plan implementation/ or health plan implement*.ti,ab. or exp program development/ or program development.ti,ab. or exp quality indicators, health care/ or quality indicator*.ti,ab. or implementation strateg*.ti,ab. or exp program evaluation/ or program evaluat*.ti,ab. or exp information dissemination/ or information disseminat*.ti,ab. or information distribut*.ti,ab. or exp organizational innovation/ or organi?ational innovat*.ti,ab. or organi?ational chang*.ti,ab. or exp diffusion of innovation/ or exp models, educational/ or exp models, organizational/ or Quality improvement/
AND
Exp Quality of health care/ or quality*.ti,ab. or program evaluat*.ti,ab. or improv*.ti,ab. or chang*.ti,ab.
Limitation
not ((exp child/ or exp adolescent/) not exp adult/)

yr="2000 -Current"

PsycINFO
Exp Palliative care/ or palliat*.ti,ab. or terminal care.ti,ab. or Exp hospice/ or hospic*.ti,ab. or end of life care.ti,ab. or comfort care.ti,ab. or supportive care.ti,ab.
AND
health plan implement*.ti,ab. or exp Program development/ or program development.ti,ab. or exp Program evaluation/ or program evaluat*.ti,ab. or quality indicator*.ti,ab. or implementation strateg*.ti,ab. or Information dissemination/ or information disseminat*.ti,ab. or information distribut*.ti,ab. or organi?ational innovat*.ti,ab. or exp Organizational change/ or organi?ational chang*.ti,ab. or diffusion of innovation.ti,ab. or Organizational development/ or organi?ational develop*.ti,ab. or Organizational learning/ or organi?ational learning.ti,ab.
AND
Exp Quality of services/ or quality*.ti,ab. or exp Program evaluation/ or program evaluat*.ti,ab. or improv*.ti,ab. or chang*.ti,ab. or Evaluation/ or evaluat*.ti,ab. or Professional standards/ or professional standards.ti,ab. or Best practices/ or best practices.ti,ab.
Limitation
Yr=”2000-current
