# Supplementary material for: Implementation of improvement strategies in palliative care: an integrative review
Source: Implement Sci. 2015 Jul 26;10:103. doi: 10.1186/s13012-015-0293-2 (PMC4515317; doi:10.1186/s13012-015-0293-2)
Supplement: Additional file 2: — Description studies. Summarized description of the methodology, setting and country, number and type of participants, the implementation strategy, and the impact of the strategy of each individual study. (PDF 182 kb) [file 13012_2015_293_MOESM2_ESM.pdf]

Appendix 2: Description studies

Source	Study methodology	Setting / country	Participants in intervention (n)	Description of strategy	Results
**Single intervention studies**					
**Large scale educational methods**					
**Lecture**					
Schim, 2006	Experimental study	Hospice / USA	Administration, clergy, clerical, nurse, nursing assistant, social worker, volunteers and others (n=130)	One-hour educational session on cultural considerations adapted from the End-of-Life Nursing Education Consortium Training Materials Module.	Pre- (n=130) and post-intervention (n=107) cultural competence assessment showed that cultural competencies improved.
Ke, 2008	Experimental study	Hospital / Taiwan	Nurses (n=88)	Intervention group received a 50-minute lecture on artificial nutrition and hydration.	Pre (n=88) and post-intervention (n=88) structured questionnaires showed a significant improvement in knowledge, attitudes and behavioural intentions within the intervention group. Changes in knowledge and attitude were significantly different between experimental and control group. Changes of behavioural intentions between experimental and control group were not significant.
Ersek, 2006	Quasi experimental study	Nursing home, hospice / USA	Administrators, Director of nursing, Staff development/ educators, Social workers, Staff nurses, Coordinators, Others (n=87)	A two-day palliative care educational resource team train-the-trainer workshop was provided. The workshop consisted of 16 modules (from the philosophy of end-of-life care to ensuring quality end-of-life care) and were conveyed through didactic presentations. Participants received a manual and CD with all course information.	Pre- and post workshop teaching effectiveness was assessed through self-evaluation and showed a significant improvement in all areas. Participants assessed the course materials as comprehensive, appropriate and useful for clinical practice. Confidence in teaching end-of-life content increased significantly for participants who used the course materials to prepare and present inservice.

Source	Study methodology	Setting / country	Participants in intervention (n)	Description of strategy	Results
**Study day**					
Carr, 2003	Quasi-experimental study	Setting unknown / UK	Nurses, physicians, physiotherapists, occupational therapists, pharmacist (n=on average 18 per session)	4 half-day study days were conducted. For each session, 2 cases were identified by charge nurse and a team member presented each case. Discussion was used for problem solving. Sessions were facilitated by 2 or 3 members of the steering committee.	Average pain scores decreased from pre-intervention 2.9 (n=30) to 2.0 (n=28) as there was an increase in pain assessment, discussion about pain and requests for transcutaneous electrical nerve stimulation (TENS) machines. Decrease in referrals to acute pain team. Post intervention evaluations showed that sessions on chronic pain were considered more useful compared to session on acute pain. All participants indicated that learning with other professions and gaining new knowledge and ideas was particularly valuable.
Dryden, 2009	Quasi-experimental study	Hospital, Hospice & Nursing home, and Community & Day care / UK	Health care assistants and social care officers (n=52)	A study day (including lecture and group work) was used to teach health care assistants how to help patients cope with the symptoms of advanced disease.	Post-intervention evaluation forms (n=47) and pre and post-test self-report questionnaires (n=42) showed improvements in knowledge and self-confidence.
**Small scale educational methods**					
**Role play**					
Back, 2007	Quasi experimental study	Unknown	Oncology fellows (n=115)	A biannual, 4-day residential communication skills workshop for 20 fellows per workshop was provided. Content of the workshop was focused on: 1) developing a relationship and dealing with uncertainty, 2) giving bad news, 3) discussing transition to palliative care, and 4) discussing DNR orders. The workshop was organized around 5 simulated patients (actors) and most of teaching done in small groups of 5 participants and 1 facilitator.	A preretreat questionnaire, and 2 preretreat and 2 postretreat patient encounters (n=106 pairs) showed improved communication skills in the postretreat encounters.

Source	Study methodology	Setting / country	Participants in intervention (n)	Description of strategy	Results
Hales, 2008	Quasi-experimental study	Hospital / Canada	Intensivist, nurse, social worker, respiratory therapist, clinical nurse educator (n=36)	Teams of 3-6 professionals rotated through six 45-minute stations, enacting scenarios with trained actors.	Pre and post-test questionnaires (n=18) showed improved ethical and legal knowledge and confidence in communication.
**Interactive education**					
Bruneau, 2004	Quasi-experimental study	Hospital / UK	Nurses (n=18)	Two sessions of 2-hours each. First session explored participants' knowledge and experience of stress. Second session reviewed the issues explored in the first session and provided an opportunity to practice relaxation techniques.	Pre and post-test questionnaires and interviews (n=18) indicated no conclusive evidence that the stress-reduction programme had any impact on participants' stress levels or their capacity for coping with stress.
Cooke, 2004	Quasi-experimental study	Hospital / USA	Nurses, psychologists, physical therapists, occupational therapists, social workers, physicians, administrators (n=unknown)	1-hour, rotating, monthly program of case presentations and analysis to assist clinical nurses in translating research and ongoing knowledge into practice: facilitator introduced purpose of presentation, staff nurse presented the case study. 5-minute presentation about evidence based practice was given, 3 team members presented for 10 minutes on relevant nursing issues using EBP from the literature, a 20-30 minute discussion followed.	Pre-post intervention evaluation revealed that confidence in knowledge increased by almost one point on a five-point scale.
**Outreach visit**					
Newton, 2009	Quasi-experimental study	Community care, Hospital, Hospice & Nursing home / UK	Nurses (n=33)	Implementation of the preferred place of care document, including a 1-3 hour face-to-face educational contact with each study site, prior to the commencement of the preferred place of care document.	Assessment of preferred place of care document and periodic questionnaire showed that 85% of the nurses using the document stated that it had affected their practice, because it helped raise awareness. The document was initiated later in the patient trajectory than recommended. 71% of the patients during the study period died at their PPC.

Source	Study methodology	Setting / country	Participants in intervention (n)	Description of strategy	Results
**Computer facilitated education**					
Ersek, 2008	Quasi-experimental study	Home care, Nursing home, hospice & hospital / USA	Nursing assistants (n=65)	Six hours of nursing assistant computerized education programme was used over a six week period, which was based on the palliative care educational resource team programme and the core curriculum for the hospice and palliative nursing assistant.	Evaluation questionnaire, and pretest-posttest end-of-life care knowledge exam and self-evaluations showed a significantly increased knowledge and improved perceived skills in caring for patients at the end of life.
Hulsman, 2002	Quasi experimental study	Hospital / the Netherlands	Physicians (n=21)	A 4 hour computer assisted communication skills training, consisted of 4 modules. Module 1: focused on basic communication skills regarding verbal and non-verbal behaviour of physician and patient. Module 2: breaking bad news. Module 3: providing information effectively, based on two-way interaction. Module 4: dealing with patients' emotions. In each module, video examples were used to present poor and adequate communication.	Pre and post intervention videotaped patient interviews and patient satisfaction ratings showed a positive course effect on rating of the quality of physicians' performance. No course effect were found on the frequencies of physicians' communication behaviours and on the patient satisfaction rating.
Jarabek, 2008	Quasi-experimental study	Hospital / USA	Internal medical house staff (n=144)	Implementation of palliative care order set: An educational e-mail was sent which outlined each section within the order set with links to internal and external web sites referencing palliative management of pain, sedation, delirium, secretions, dyspnoea, code status, nutrition and fluids. The e-mail was only sent once, when the order set was introduced.	Pre-intervention (n=97) and post-intervention (n=76) evaluation showed a significant increase in the percentage of residents comfortable with regard to the four aspects of symptom management.
Smith, 2010	Quasi-experimental study	Various / UK	Nurses (n=30)	Six week, online communication and end-of-life assessment skills training.	Pre and post-test self-assessment questionnaire, post-intervention candidate evaluation, and post-intervention moderator evaluation showed improvements in participants' confidence.
**Non education interventions**					
**Process mapping**					
Taylor, 2007	Quasi-	Nursing	Manager, care	A meeting was arranged with members of the	After six months, the process was

Source	Study methodology	Setting / country	Participants in intervention (n)	Description of strategy	Results
	experimental study	home / New Zealand	manager, senior nurse, pharmacist, LCP facilitator, LCP consultant pharmacist (n=unknown)	interdisciplinary team to map the process of care in order to implement the Liverpool Care Pathway. The second part of the meeting involved looking at the bottlenecks.	remapped, demonstrating a distinct reduction in bottlenecks and improved pre-emptive prescribing.
**Feedback**					
Velikova, 2004	Experimental study	Hospital / UK	Physicians (n=28)	Patients were allocated to intervention group (completion of touch-screen health related quality of life questionnaire and feedback of results to physician), attention-control group (completion of touch-screen health related quality of life questionnaire, without feedback to physician), and control group (no touch-screen measurement of health related quality of life).	Physicians referred explicitly to the health related quality of life data in only 64% of the encounters. However, a larger proportion of patients in the intervention group showed clinically meaningful improvement in HRQL compared with patients in attention-control and control groups. Routine use of HRQL information during the encounters had an impact on physician-patient communication without prolonging the interviews. Chronic nonspecific symptoms were discussed more frequently.
**Multidisciplinary meetings**					
Lilly, 2000 & Lilly 2003	Quasi experimental study	Hospital / USA	Physicians, nurses (n=54)	In the preintervention period, formal family meetings were usually held after the provider team had reached consensus that restoration of function or survival was unlikely. In the intervention period, an initial formal multidisciplinary meeting with the patient, family or both was held within 72 hours after admission to the ICU. Purpose of the meeting was: 1) review medical facts and options for treatment; 2) discuss patients' perspectives; 3) to agree on care plan; and 4) to agree on criteria by which the success or failure of this	Preintervention (n=134) compared to intervention (n=396) demonstrated a significant increase in physician-led meetings, length of stay in the ICU was reduced from 4 days to 3 days (after 4 years, this was still 3 days) and there was a decrease in unadjusted overall mortality (p=0.02) and a trend toward reduced mortality during the ICU stay. The rate of provider non-consensus days decreased

Source	Study methodol ogy	Setting / country	Participants in intervention (n)	Description of strategy	Results
				care plan would be judged. Weekly multidisciplinary case reviews were held to ensure that all relevant patients had sessions.	from 65 days per 1,000 patient-days to 4 days per 1,000 patient-days. The rate of family non-consensus decreased from 171 days 1000 patient-days to 16 days per 1000 patient-days.
**Multiple intervention studies**					
**Education with different strategies**					
Kruse, 2008	Experiment al study	Hospital / USA	Nurses (n=81)	Control group received an article to read. Intervention group 1 received a 3-hour didactic presentation, including case study, presentation and discussion. Intervention group 2 received 3-hour didactic presentation + 4-hour bedside clinical experience at a local hospice.	Pre and post-test questionnaire showed no significant differences in perception of end-of-life care were found between control and intervention groups.
Razavi, 2003 & Lienard, 2006 & Lienard, 2007 & Merckaert 2008	Experiment al study	Setting unknown / Belgium	Physicians (n=72)	Physicians received a basic communications skill training (2-hour lecture and 17-hour small group role-playing sessions). Subsequently, physicians were randomly selected to receive 6 3-hour workshops (same program as basic training) or were placed on the waiting (consolidation workshop started 2 months later).	Consolidation workshop improved communication skills. Simulated interviews (n=62) showed: significant increase in open and open directive questions and utterances alerting patients to reality. Significant decrease in premature reassurance. Patient interviews (n=59) showed: significant increase in acknowledgements, emphatic statements, educated guesses, negotations. No significant correlations were observed at baseline between pre-post consultation evolution of patients' anxiety and physicians' assessment. No significant effect group by time on changes in patients' and relatives' anxiety following a three-person consultation was observed. No

Source	Study methodology	Setting / country	Participants in intervention (n)	Description of strategy	Results
					significant changes were noted in physicians' ability to detect relatives' distress . However, there was an effect of the workshops on physicians' ability to detect patients' distress when they are accompanied by a relative.
Razavi, 2002 & Delvaux, 2004	Experimental study	Hospitals / Belgium	Nurses (n=116)	Nurses of the intervention group participated in a three week communication skills training (one week for each of the three consecutive months). The programme included theoretical information (30h), experiential exchange (case presentations) and role-playing exercises (75h). Each subject participated in 4 role-play exercises. Nurses in the control group, were trained after having completed all their assessments.	Nurses completed recorded interviews (one role-play and one clinical interview) at baseline (n=114), one week after the end of training (n=111) and three months later (n=110). Results show that professional empathy increased (professionals use more emotional words after training). Compared to control, trained nurses reported positive changes on their stress level and attitude (both significant). Significant effect was found for communication skills, but no training effect was observed on nurses' satisfaction levels. However, a positive training effect was found on patients' satisfaction levels.
Wilkinson, 2008	Experimental study	Community nursing service, hospices / UK + Ireland	Nurses (n=170)	Nurses completed an audio-taped nursing assessment after which they were randomly included in a) control group, or b) intervention group. The intervention consisted of a 3-day communication skills training, which included a didactic overview, exploration of communication behaviours, interactive demonstrations, facilitated role-play, constructive feedback, discussion, consolidation materials (handouts, references, etc.).	Audio-taped nursing assessment before (85 in control arm, and 82 in intervention group) and after (82 in control arm, and 72 in intervention group) the training demonstrated significant improvement in communication skills and coverage and confidence of nurses. Patient satisfaction was also greater in the intervention group.
Bailey, 2005	Quasi-	Hospital /	Physicians, nurses	I hour educational sessions in 3 cycles of 16	Pre and post-test record review

Source	Study methodology	Setting / country	Participants in intervention (n)	Description of strategy	Results
	experimental study	USA	and other staff (n=unknown)	weeks each were conducted to teach staff communication skills and how to identify patients in need of end-of-life care and implement care plans. After these educational sessions, more informal case-based teaching was undertaken. Verbal reminders and laminated pocket cards were used to assist and remind staff to identify patients who were near the end of life. A set of comfort care plans was also developed and condensed into a laminated pocket card. New nursing and pharmacy policies were issued to ensure the implementation of the care plans.	indicated increased documentation and care plans for 12 of the 13 end-of-life symptoms. Significant increase in number of symptoms documented, number of care plans, availability of opioid pain medication, Do-not-resuscitate (DNR) orders, palliative care consultations and use of restraints. Significant decrease in resuscitation attempts. Non-significant decrease in withdrawal of nasogastric tubes.
Betcher, 2010	Quasi-experimental study	Hospital / USA	Nurses (n=8)	45 minute lecture followed by role-play with simulation and discussion to improve communication skills of nurses.	Pre and post-test caring efficacy scales indicated improved confidence in the ability to portray caring attitude and helped to improve communication skills with patients and their families.
Bravemen, 2001	Quasi-experimental study	Hospice, home care / USA	Unknown	1st intervention: Education of staff about pain management via two 1-hour in-service training sessions of physical, psychosocial, social and spiritual manifestations of pain and pain interventions. This education consisted of an introductory lecture, discussions and role-playing. A laminated pocket-card and checklists were developed which included the most important information, and all staff were given a set of guidelines. A pain expert was created at each site. 2nd intervention: weekly pain newsletter	Pre and post-test competency assessment and chart audits indicated for the 1st intervention: Some improvements in documentation. Little improvements in staff competency. And for the 2nd intervention: great improvement in staff competency.
Bylund, 2010	Quasi-experimental study	Hospital / USA	Physicians (n=36)	Six 3-hour training sessions, consisting of didactic presentation to review evidence and introduce communication skills, a demonstration video, small group role-play to practice skills, and feedback related to their role-play sessions.	Pre and post-test assessment of video recorded consultations (n=112) showed significant improvements in two communication skill sets (establishing consultation framework and checking). Some

Source	Study methodology	Setting / country	Participants in intervention (n)	Description of strategy	Results
					change (though limited) in questioning, empathic communication and information organization skills. No improvement in shared decision making skills.
Favre, 2007	Quasi experimental study	Setting unknown / Switzerland	Physicians, nurses (n=10)	A 2-day communication skills training consisted of case-history discussion, analyses of videotaped interviews with simulated patients, and structured role-play. Follow-up consisted of four to six individual supervisions. After 6-months, another half-day training session including role-play (videotaped interviews) took place.	Observer-rated videotaped interviews before and after the course showed significantly increased overall defensive functioning, significant decrease of immature defences, significant decrease in borderline defence and, on the individual level, the overall defensive functioning improved for 8 out of 10 clinicians.
Fallowfield, 2001	Quasi experimental study	Hospital / UK	Nurses (n=129)	A two-day communication skills training was given to small groups (12 persons per session). Participants worked on specific skills in small groups of four or less, using video review of role-play with standardized patients. Interactive group demonstrations, small group discussion of prepared video materials and a resource file of selected key reading provided a conceptual framework and expansion of participants' knowledge of the literature.	Before and 3-months after the course (n=92), nurses filled in a questionnaire. Posttest data showed that nurses reported significantly greater confidence in handling 14 common communication problem areas in cancer and in 8 different areas of teaching. Three months post-course 91% reported changing their own teaching practice and 85% had initiated new communication skills teaching.
Finset, 2003	Quasi experimental study	Setting unknown / Denmark, Finland, Iceland, Norway and Sweden	Physicians (n=219)	A communication skills course, consisting of three modules. Module 1: three seminars of 3 hours at two week intervals about interview techniques, psychological defence mechanisms and crisis reactions, using instructional videos and self-study materials. Module 2: a boarding course lasting for 3 days, including experiential learning in the form of role-play, which was videotaped to stimulate discussion. Module 3: three seminars of 3 hours aimed at	Physicians filled in a questionnaire before, at the completion, and 2 to 6 years after course completion (n= 155). 94% of the physicians reported high degree to fairly large extent of satisfaction of the course content. The course improved all communication learning needs, except applying knowledge of defence mechanisms, meeting

Source	Study methodology	Setting / country	Participants in intervention (n)	Description of strategy	Results
				how doctors experienced their work with cancer patient and testing new skills.	depression in patients, meeting withdrawal in patients, and meeting suicidal patients. Participants described that communication in challenging situations is easier than before the course.
Fischer, 2007	Quasi experimental study	Hospital / USA	Internal medicine interns (n=51)	A 3-hour communication skills workshop was provided. The workshop consisted of group discussions, a brief lecture, videotape review, and role-playing.	Pretest (n=43) and posttest (n=29) survey showed an increase in overall knowledge, bad news subscore, and advance care planning subscore. There was also an increase in participants' confidence in ability to discuss advance care planning and limitations of treatment, delivering bad news and handling emotional responses from patients. There was no significant differences in attitude before and after the workshop.
Furman, 2006	Quasi experimental study	Hospital / USA	Physicians (n=8)	Participants attended a morning report consisting of both didactic training and three-person role-played discussion in order to improve discussion of advance directives.	Pre (n=44) and post (n=35) intervention chart audit, showed that 32% of the patients had a documented advance directive discussion before intervention compared to 34% after the intervention. Results were not statistically significant.
Gueguen, 2009	Quasi-experimental study	Hospital / USA	Physicians, nurses, nurse practitioners, physician assistants (n=40)	Didactic presentation, including exemplary video clips, followed by role-play with discussion and feedback to improve communication skills. Participants were also given an educational booklet.	Anonymous, post-test evaluation forms showed that the confidence in conducting family meeting increased significantly from retrospective pre-test evaluation.
Hall, 2007	Quasi-experimental study	Palliative care unit / Canada	Physicians, nurses (n=17)	Four-hour education sessions for nurses and case study and role-play were used by physicians to review communication strategies, shared decision-making and care planning concepts. An advanced practice nurse was available on the unit every day when the	Daily logs of activities and satisfaction, pre and post-test questionnaires, interviews, and focus groups indicated improved communication between physicians and nurses and the increased

Source	Study methodology	Setting / country	Participants in intervention (n)	Description of strategy	Results
				pilot began to support staff with the change process.	assessment responsibility of nurses reduced physician time on the ward.
Kinnane, 2009	Quasi-experimental study	Hospital / Australia	Volunteerd and volunteer coordinators (n=8)	3-day training, including classroom and group activities (e.g. role-play) about the service, communication skills, support and self-care.	Pre-post intervention (n=8) showed improvements in: communication skills, offering support, knowledge of available service, confidence. Most valued aspect of training: role-play, learning communication skills, information of the services.
Quinn, 2008	Quasi-experimental study	Hospital, Nursing home, General practitioner, hospice / Australia	Allied health care professionals, physicians, nurses, personal care attendant (n=495)	Nine sessions presented by specialist palliative care clinicians. Sessions consisted of didactic lectures, question and answer sessions, workshops and panel/ case discussions. Participants were also given educational material.	Pre and post-test questionnaire, post-test evaluation, and a focus group showed a significant improvement in ability to identify palliative care patients, relationship and roles, providing support services, communication skills, symptom and pain management, legal and ethical issues, spiritual and cultural aspects and grief and bereavement.
Reymond, 2005	Quasi-experimental study	Primary care / Australia	GP, nurse, nurse assistant, personal care worker, indigenous health worker, respite care worker, diversional therapist, Physiotherapist, social worker, pastoral worker (n=149)	3-hour workshop consisting of introductory didactic teaching on participant nominated topics, small group case management discussion, and a session devoted to psychosocial and counseling inputs.	Pre and post-workshop questionnaires (n=116) showed that GPs' confidence increased significantly in knowledge and skill levels in managing common symptoms (nociceptive tissue pain, neuropathic pain, dyspnoea, constipation, and delirium). Other primary health care workers had a significant improvement in rated knowledge of treatment of pain and dyspnea (constipation was not significant), and rated skill level was statistically increased for pain management in dementia. Their confidence in developing management plans and

Source	Study methodology	Setting / country	Participants in intervention (n)	Description of strategy	Results
					communicating with patients also increased significantly.
Sullivan, 2005	Quasi experimental study	Setting unknown / USA	Physicians, nurses, social workers, pharmacists, ethicists, and other health care professionals (n=149)	Palliative Care Education and Practice was delivered in two, one-week, full-time, on-site sessions, separated by 6-months that included an interim distance-learning component. Session 1 focused on fundamental clinical and educational aspects of palliative care. Session 2 included experiential learning in clinical and teaching skills and training in leadership and organizational change. During the interim period, participants worked on individual projects, developing a clinical or educational program and contributed to weekly e-mail case discussions.	Self-administered questionnaires before (n=149) and after (n=113) the training demonstrated statistically significant improvements on nearly all measures, including knowledge skills and confidence levels.
Sutherland, 2007	Quasi experimental study	Hospital / Australia	Physicians (n=93)	A 4,5 hour workshop on breaking bad news started with the theoretical underpinnings of communication skills. Subsequently, group based activities, including role-play, and discussions were used to practice and understand how evidence-based theory translated to practice. Participants received a booklet with guidelines, information, a glossary and bibliography.	Pre (n=54) and post (n=34) workshop questionnaires demonstrated significant improvements in confidence and in 3 out of 8 items assessing subjective reports of clinical behaviour.
Weissman, 2000	Quasi experimental study	Long term care facilities / USA	Unknown	Four educational programs, one every 3 months (20 contact hours), included lectures, demonstrations, small group case-based discussions, role-play and panel discussions to teach and practice pain assessment. Participants received a recourse pack that included content outline, overheads and supporting material (national pain management treatment guidelines, sample pain assessment tools, and pain management policies).	Assessment of pain policies before and chart review after the training demonstrated improved pain practice: at baseline 14% of facilities had >51% of the indicators in place; at conclusion 74% of facilities had >51% of indicators in place.
Weissman, 2001	Quasi-experimental study	Long term care / USA	Nurses, different levels of management	Four educational workshops, consisting of lectures, group workshops, role-play exercises and case studies, spread over a one-year	Pre-test needs assessment and post-test chart review indicated improved knowledge and

Source	Study methodology	Setting / country	Participants in intervention (n)	Description of strategy	Results
			(n=unknown)	period.	confidence in participants' own practice. On the organizational level, an improvement on number of target indicators that were reached was reported.
Wilkinson, 2002	Quasi-experimental study	Various settings / UK	Nurses (n=308)	A 3-day communication skills training was delivered over a 3-month period. The training consisting of 26 hours and included attitudes to cancer, communication with patients, relations and colleagues, nonverbal communication skills, assessing psychological distress, strategies for handling difficult situations, and stress and survival in cancer nursing settings. Teaching methods included audiotaped feedback, demonstration videos and role play discussions.	Pre and post course tape recorded nursing assessment, questionnaire (pre course only) and written self critique (post course only) demonstrated significant improvement on all 9 individual areas of the communication skills assessment.
Wilkinson, 2003	Quasi-experimental study	Hospital, hospice, community care / UK	Nurses (n=108)	3-day communication skills training, using a teaching pack, verbal and written feedback on audio recording of nursing assessment, video and audio demonstration tapes, didactic sessions, discussions, and role-play with feedback.	Pre and post-test assessment of audio tape recording (n=101) showed significant improvements in assessment skills, confidence in communication skills and perceived confidence in teaching communication skills.
Yamagishi, 2009	Quasi-experimental study	Hospital / Japan	Nurses (n=81)	Educational session about artificial hydration therapy for terminally ill cancer patients, which consisted of a 10-minute lecture, four interactive 60-minute seminars and discussion.	Pre and post-test (n=76) knowledge, confidence, self-reported practice, and overall evaluation showed that knowledge and confidence scores significantly increased.
**Mixed interventions**					
Butow, 2008	Experimental study	Hospital / Australia	Physicians (n=30)	Two groups of physicians, one intervention group and one control group. The intervention group received a booklet summarizing the evidence underpinning the recommendation before the workshop. Then they underwent a 1.5-day intensive face-to-face communication skills training with 3-6 participants each time,	Simulated patient interviews and questionnaire were administered to all physicians, including those from the control group. The intervention group was also assessed immediately after the training, and at 6 and 12 months. Results show

Source	Study methodol ogy	Setting / country	Participants in intervention (n)	Description of strategy	Results
				including presentation of principles, a DVD modelling ideal behaviour and role-play practices, followed by four 1.5 hour follow-up video-conferences incorporating role-play. The participants conducted the role-play with actors, while the facilitator joined in through video-conference.	that physicians displayed more creating environment and fewer blocking behaviours at follow-up, but were not significant. Physicians valued the training highly, but did not report substantial reduction in stress and burnout.
Curtis, 2011	Experiment al study	Hospital / USA	Physicians, nurses, respiratory therapists, social workers and spiritual care workers (n=unknown)	Clinician education about palliative care using grand rounds, workshops, academic detailing and video presentations. Training of champions for palliative care. Identification of ICU-specific barriers to palliative care. Feedback of ICU-specific family satisfaction data. Implementation of system supports such as the palliative care order forms.	Pre and post-test patient, caregiver, and staff questionnaires showed that the intervention was not associated with significant changes in any of the family-assessed or nurse-assessed outcomes. Also no changes in length of stay in ICU before death or change in time from admission to withdrawal of life-sustaining measures (mechanical ventilation).
Fallowfield, 2002	Experiment al study	Hospital / UK	Oncologists (n=160)	Oncologists were randomly assigned to four groups: 1) written feedback followed by course, 2) course only, 3) written feedback only, 4) control (no feedback and no course). Feedback consisted of comprehensive written feedback on videotaped consultations based on analysis of the doctor's communication skills, patients' satisfaction scores, comments after consultation, doctor's rating of patients' distress and understanding of information. The 3-day course consisted of reviewing assessments and small group role-play simulations in order to improve oncologists' communication skills.	Differences between baseline assessment (n=320) and 3 months after the course (n=320) showed a significant improvement in the number of focused and open questions, expressions of empathy, and appropriate responses to patients' cues for oncologists who had been on the course compared to those who had not. There was little evidence for the effectiveness of written feedback.
Jenkins, 2002	Experiment al study	Hospital / UK	Physicians (n=93)	See fallowfield, 2002	Pre and post intervention questionnaire and videotaped patient interview showed significantly improved attitudes and beliefs toward psychosocial issues compared with controls.

Source	Study methodology	Setting / country	Participants in intervention (n)	Description of strategy	Results
Shilling, 2003	Experimental study	Hospital / UK	Oncologists (n=160)	See Fallowfield 2002	Patient and clinician satisfaction were measured before (n=464) and 3 months after (n=439) the course and before (n=491) and after (n=422) the course for the control group. Despite the measureable changes in clinicians' behaviour, the communication skills training had no significant effect on either patient or doctor satisfaction with the consultation.
Fallowfield, 2003	Experimental study	Hospital / UK	Oncologists (n=160)	See Fallowfield, 2002<br>This study describes a 12 month follow-up.	12 month follow-up assessments of videotaped patient interviews, showed that the effect recorded after 3 months was still in effect after 12 months. There were fewer interruptions evident and summarising of information increased, responses to patient-led cues improved while the number of inappropriate responses decreased.
Hanson, 2005	Experimental study	Nursing home / USA	Directors of nursing, nurses, nursing assistants, social workers, staff development coordinators, MDS coordinators, administrators, Activities directors, Nurse practitioner or physician assistant, other nursing home staff (n=unknown)	One-day conference about hospice enrollment and service, pain management, advanced care planning, communication, and quality improvement techniques. Monthly on-site education about quality improvement techniques. These sessions were videotaped to facilitate delivery to evening and night staff. The palliative care leadership team participated in monthly strategy meetings to design and implement stepwise organizational changes. They received and responded to performance data recorded at baseline, month 3 and month 6.	Pre and post-test (n=41) knowledge test and pre and post-test chart reviews indicated improved knowledge and confidence to provide good quality palliative care. Increased hospice enrollment, number of pain assessments, non-pharmacological treatment and in-depth discussions about end-of-life care with residents. The prevalence of pain and the use of pain medication did not change.

Source	Study methodology	Setting / country	Participants in intervention (n)	Description of strategy	Results
Roila, 2004	Experimental study	Hospital / Italy	Oncologists (n=unknown)	Group 1: Diffusion of guideline, group 2: Diffusion of guideline + audit and feedback, group 3: Diffusion of guideline + audit and feedback + educational outreach visit.	Monitoring of the antiemetic prescription showed that a simple diffusion of guideline was not very effective. Simple diffusion of guideline + audit and feedback also resulted in poor results. Combination of diffusion of guideline, audit and feedback, and education outreach visit had a positive impact on antiemetic prescription, but inferior to what was expected.
Okon, 2009	Quasi-experimental study	Hospital / USA	Nurses (n=364)	A hospital-wide, computer-based clinical documentation of pain system (including a delayed reassessment alert intervention) was implemented using a detailed printed explanation, brief group training sessions and feedback reports.	Pre and post-test chart audits revealed a reduced delay in pain reassessment time to resolution of severe pain and hospital-wide administration of naloxone.
Boakes, 2000	Quasi-experimental study	Primary care / Australia	GPs (n=22)	GPs attended weekly patient case conferences to review their own practice, they were asked to keep a diary of patient related activities, they were also required to take part in the hospice team's normal on-call roster, they participated in tutorial-type educational sessions (covering: pain management, symptom control, oncology, and psychosocial and ethical issues in palliative care), and they held a weekly discussion with a mentor.	Pre and post-intervention questionnaires indicated statistical significant improvement in the GPs perceived level of knowledge, skills and confidence in providing palliative care.
Bookbinder, 2005	Quasi-experimental study	Hospital / USA	Nurses (n=unknown)	To introduce a care pathway, consisting of interdisciplinary care path, nurses' daily flowsheet, and a standardized physician order sheet, nurses received educational session about end-of-life care and house staff and physician assistants received a case-based teaching round. On each study ward, a nurse leader acted as liaison to the project. Evaluation tools were used to assess and feedback the progress made.	Pre and post-intervention chart abstraction (n=101;156), process audit, and knowledge questionnaire (n=138) indicated that the mean number of symptoms assessment increased significantly; Significant decrease in the number of problematic symptoms identified and interventions employed in the palliative care unit; Significant

Source	Study methodology	Setting / country	Participants in intervention (n)	Description of strategy	Results
					increase in the number of inpatient consultations; Increased discussion of patient's goals of care and patient preferences; Increased use of palliative care in vocabulary of physicians; Increased collaboration between hospice and hospital. Morphine infusion and DNR orders were more likely in study units. Although mean knowledge scores improved, no significant changes were found. Number of patients placed on care pathway: 9 of 27 in Oncology/Geriatric units, all 50 in PC unit
Dauer, 2006	Quasi-experimental study	Hospital / USA	Nurses (n=113)	Radiation safety training consisting of revised nursing radiation safety procedures, radiation safety core concepts video, interactive nurse leadership in-service training, didactic nursing staff in-service training, and improved radiation precaution information signs/labels.	Pre and post-test (n=113) assessment showed significant improvements in nurses' cognitive knowledge and attitude towards radiation safety.
Hansen, 2009	Quasi-experimental study	Hospital / Country unknown	Nurses (n=91-127)	A nurse-developed bereavement program for patients' families, collaboration with/use of a palliative medicine and comfort care team, use of preprinted orders for the withdrawal of life-sustaining treatment, hiring of a mental health clinical nurse specialist and staff education in end-of-life care.	Pre (n=91) and post-test (n=127) questionnaire showed that the perception of work environment, staff support, patient, and family support and work stress improved. Perception of knowledge and ability did not differ significantly over time.
Hills, 2009	Quasi-experimental study	Hospital / UK	Nurses (n=unknown)	Audit to assess aftercare, development of last offices policy, interdisciplinary workshops and teaching sessions (including a DVD on the principles and standards of aftercare).	Pre (n=43) and post-test (n=42) audits showed improvements in care and raised the profile of aftercare. Improvements achieved following the audit were modest.
Hockley, 2010	Quasi-experimental study	Nursing home / UK	Nurses and caring staff (n=unknown)	1-3 key champions in each service (who received 4 workshops and a 4-day training course) implemented a list of all permanent residents with prompts for monthly	Retrospective review of clinical notes before (n=95) and during the intervention (n=133) and post-intervention staff audit (n=68)

Source	Study methodology	Setting / country	Participants in intervention (n)	Description of strategy	Results
				discussions around advanced care planning, DNAR status, family and resident communication, and symptom assessment control. Staff received a 2-hour scenario-based training. Each service also had an outreach visit from the project facilitator every 10-14 days.	showed that documentation of DNAR instructions rose from 15% to 72%. Evidence of conversations about advanced care planning rose from 4% to 53%. The use of the adapted Liverpool Care Pathway rose from 3% to 30%. Hospital deaths reduced from 15% to 8% and number of hospital bed-days reduced by 38 %. Improvement in staff confidence in addressing psycho-social and emotional needs and talking with patients and relatives about dying.
Jacobs, 2002	Quasi-experimental study	Hospital / USA	Physicians and nurses (n=unknown)	To improve end-of-life care physicians and nurses were given biannual feedback through a palliative care report card. These reports were also presented to the medical director. Other interventions included palliative care social work consultations and educational sessions about symptom management, psychological/social concerns, bioethical and legal issues.	Medical record review (n=194), family interviews (n=133), and a physician survey (n=8) showed significant improvement in the management of respiratory symptoms, but no changes in other indicators. However, the project led to some changes being initiated in institutional policies and clinical management.
Kinley, 2004	Quasi-experimental study	Palliative care unit / UK	Nurses and medical staff (n=33)	Oral care documentation, including an assessment tool, oral care plan, and protocol of care were implemented. Staff received a refresher training on oral care, including feedback from baseline audit, teaching about oral care, discussion about products, demonstration of oral care, discussion and a video. An information leaflet (in the form of a bookmark) was also developed.	Pre (n=50) and post-test (n=47) retrospective review of patient records and pretest-posttest staff knowledge questionnaire (n=23) showed improvements in awareness and knowledge.
Keay, 2003	Quasi-experimental study	Nursing home / USA	Physicians (n=12)	A half-day, educational outreach programme based on a curriculum for educating nursing home physicians in palliative and hospice care. Programme included audit & feedback, use of opinion leaders and review of guidelines.	Pre and post test questionnaires and terminal care audit forms showed that the intervention was associated with significant improvements in terminal care

Source	Study methodology	Setting / country	Participants in intervention (n)	Description of strategy	Results
					outcomes: improved pain control, better control of shortness of breath, better control of uncomfortable symptoms, more attention to hygiene and bereavement. Increased use of WHO class 3 medications. Decrease in WHO class 1 medication. Number of unexpected deaths dropped from 36% to 5%. More documentation of comfort measures. Better documentation of residents' advance directives.
Lankshear, 2010	Quasi-experimental study	Hospital / Canada	Physicians (n=96)	Full-day orientation for clinician mentors, regional education session for clinicians and administrators, presentations at rounds and department meetings were used to launch the project. Clinician mentors held educational information seminars for small groups and individual physicians. Update on project was a standing agenda item at regular meetings in the centers. Monitoring and feedback, including monthly conference calls and monthly quality assurance reports on both center and individual physician level, were used to facilitate ongoing dialogue and motivate physicians to improve documentation of cancer stage.	Pre (n=52) and post-test (n=42) questionnaire indicated that documentation of cancer stage improved on both organizational and provincial levels.
Lyon, 2007	Quasi-experimental study	Care homes / Australia	Nurses and medical staff (n=unknown)	An audit-feedback approach was used to implement 1) Monthly meetings, 2) External assistance: 2.1) assistance with the development of an implementation strategy and assistance with system changes, 2.2) two-day training and additional one-to-one assistance with an experienced mentor, 3) Information sessions on advanced care planning including introductory discussion and information kits for doctors who were not able	Pre and post-implementation audit of residents' files (n=46) indicated improvements in documented evidence that the resident has been involved in advanced care planning, significant others have had the opportunity to be involved in advanced care planning and there is evidence of ongoing assessments. Pre- and post-

Source	Study methodology	Setting / country	Participants in intervention (n)	Description of strategy	Results
				to attend the sessions, and 4) Documenting and implementing guidelines. During the course of the project, regular but optional support meetings were used to discuss personal encounters. At the end of the project, a short session about advanced care planning was added to a mandatory study day.	implementation audit of staff (n=6) indicated that advanced care plans are more regularly completed and implemented after receiving training.
McCormick, 2010	Quasi-experimental study	Hospital / USA	Social workers (n=35)	Social workers participated in clinician education, local champion development and feedback of family satisfaction data.	Pre (n=66) and post-test (n=60) family satisfaction questionnaire and pre and post-test (n=15) questionnaire for social workers indicated decreased family satisfaction (not significant). Increased years as a social worker had a significant and positive effect on family satisfaction and increased caseload had a significant and negative effect on family satisfaction with social workers. Significant increase in 4 of the 14 reported activities: discussing spiritual/religious needs with the family, talk with family about touching their loved one, talk with family about disagreements in the plan of care, and assuring the family that the patient would be kept comfortable. No improvement in social workers' satisfaction with meeting families' needs or family ratings of social workers.
Mirando, 2005	Quasi-experimental study	Hospital / UK	Nurses, physicians, housekeeping staff, porters, administrative and clerical staff	A care pathway was introduced to 12 clinical areas of a hospital via multidisciplinary training, audit and feedback and reminders and support from the project nurse.	Medical record audit, monitoring variances, and evaluation of training and field notes showed a 20% increase in information-sharing, documentation of do not resuscitate orders, prescription of

Source	Study methodology	Setting / country	Participants in intervention (n)	Description of strategy	Results
			(n=unknown)		medications, recording of vital signs being discontinued, discontinuation of nonessential oral drugs, and communication with the family. Following each training session, a short questionnaire showed that the confidence of nurses to prompt a review of clinical situation improved over time.
Monteleoni, 2004	Quasi-experimental study	Hospital / USA	Unknown	A palliative care team was established, this team instituted 4 educational programmes: 1) a geriatrician gave a medical grand round. The other 3 programmes involved: rotation of 12 sessions on pain management consultation, participation in presenting modules of the EPEC curriculum, and spend one month on geriatrics rotation.	Chart review showed that the total number of tubes inserted and the number of tubes placed in patients with dementia both decreased significantly. No reduction in proportion of tubes placed in patients with an advance directive refusing artificial nutrition.
Morgan, 2010	Quasi-experimental study	Hospital / UK	Nurses and other staff (n=unknown)	Before the implementation of an end-of-life care pathway, regular informal meetings were used to teach professionals the principles of the pathway. Champions, continued education and feedback were used during the implementation phase.	A post-intervention audit (n=20) showed that the pathway is being used during decision-making and has facilitated improvement in the quality of End-of-Life Care, but documentation of bereavement care that was present pre-implementation was absent after implementation.
Reynolds, 2004	Quasi experimental study	Nursing home / USA	Unknown	Clinical leadership team visited two conferences: one pre-intervention and one post-intervention. A 3-person multidisciplinary team met monthly for 6 months with the leadership team for technical assistance. During the same 6 months, a hospice led team held on-site educational sessions. Audit and feedback of quality indicator scores were given three times, once every two months.	Residents' charts were reviewed to identify changes in advance care planning, pain management, and hospice use. This showed that four out of eight nursing homes were largely successful in improving their palliative care service.
Smith, 2009	Quasi-experiment	Hospital / USA	Nurses, nurse practitioners, and	Nursing assistants received a case-based training to practice the use of scripted	Pre and post-test knowledge questionnaires and (neuropathic)

Source	Study methodology	Setting / country	Participants in intervention (n)	Description of strategy	Results
	al study		nursing assistants (N=33)	screening language. Nurses received identical basic training with educational content on a more advanced level. Nurse practitioners received case-based didactic sessions as well as hands-on training in neuropathic pain-specific neurological examination. Following the educational sessions, all nurse participants received a pocket-sized laminated card of the treatment algorithm and assessment tool. Internet-based distant learning was used to provide education and project updates. Screening adherence was audited and fed back to the participants.	pain screening indicated that knowledge regarding neuropathic pain screening improved for all nurse groups. Screening adherence rate also improved.
Stacey, 2008	Quasi-experimental study	Cancer call center / Australia	Nurses, psychologists, and other allied health care professional (n=34)	1) A decision coaching protocol was introduced to provide a stepped approach to assessing callers needs; 2) Online decision support tutorial to be able to recognize decisional conflict, describe concepts of decision support, tailor decision support callers needs, be aware of decision aids and use the decision coaching protocol; 3) 3-hour skill-building workshop to further develop the participants skills, via: role-play using the protocol, feedback, and discussing a real patient-nurse call; 4) Training of supervisors in decision support; 5) Having the director of the helpline to address workshop participants the importance of decision support.	Pre-post intervention evaluations (n=32) showed an increase in knowledge (from 61% to 84%) and quality of decision support (from 56% to 86%).
Strumpf, 2004	Quasi experimental study	Nursing home / USA	All staff (n=unknown)	Four nursing homes formed the intervention group, and four the control group. The intervention consisted of a training of key staff (administrator, director, nurse, social worker) on five modules: introduction to palliative care, advance care planning, pain and symptom management, psychosocial support, and the palliative care delivery process. Following this training, the nurse and/or social worker became coordinators and delivered the	Assessment - which focused on advance care planning, pain and symptom control, and psychosocial concerns - demonstrated increasing number of residents with advance care plans, pain managements, higher rates of identification, assessment and treatment of symptom in the last weeks of life, and higher rates of physician visits

Source	Study methodology	Setting / country	Participants in intervention (n)	Description of strategy	Results
				same formal in-service training over a 3-month period for all disciplines. During the study, a nurse consultant was available for support on a weekly basis. In two nursing homes, an interdisciplinary palliative care team was launched that held a weekly discussion about its' residents in need of palliative care.	and x-rays.
Woo, 2011	Quasi-experimental study	Rehabilitation and convalescence unit, Geriatrics Day Hospital, Palliative Day Hospital / Hong Kong	Nurses, physicians and junior medical staff (n=118-121)	Senior geriatricians gave seminars about definitions and trajectories of various end-stage diseases, common end-stage symptoms, and their management. An end-of-life care manual was placed in each nursing station. Forms to guide symptoms monitoring, interventions, and investigations were included in patients' files. A care pathway was developed with ongoing review and consultation with staff. Forms and electronic alerts were used to inform staff about discharge options and end-of-life-care status. Workshops including role-play and interactive discussions were also conducted.	Pre (n=80 and post-test (n=89) patient symptoms and caregiver assessment showed a reduced duration of stay, fewer investigations, and fewer transfers back to the acute care hospital. Improvement in patient symptoms was achieved for pain and dizziness. Caregiver and patient satisfaction improved after the initiative. Pre- (n=118) and posttest (n=121) staff evaluations showed no significant differences regarding burnout and anxiety.
